# Quality of life and associated factors after surgical treatment of vulvar cancer by vulvar field resection (VFR)

**DOI:** 10.1007/s00404-020-05584-5

**Published:** 2020-05-19

**Authors:** Sophia Trott, Michael Höckel, Nadja Dornhöfer, Kristina Geue, Bahriye Aktas, Benjamin Wolf

**Affiliations:** 1 Department of Gynecology, University Hospital Leipzig, Liebigstr. 20a, 04103 Leipzig, Germany; 2 Division of Medical Psychology and Medical Sociology, University of Leipzig, Leipzig, Germany

**Keywords:** Quality of Life, Vulvar cancer, Vulvar field resection, Morbidity, Lymphedema, Wound healing complications

## Abstract

**Purpose:**

To investigate patient-reported quality of life (QoL) and associated factors in vulvar cancer patients treated surgically by vulvar field resection (VFR) without adjuvant radiation.

**Methods:**

We retrospectively evaluated patient-reported QoL as part of the prospective monocentric VFR trial using the 30-item European Organization for Research and Treatment of Cancer quality-of-life questionnaire (EORTC QLQ-C30) supplemented by a question assessing sexual activity. All patients had been treated by VFR and no participant had received adjuvant radiotherapy. The gynecologic cancer lymphedema questionnaire (GCLQ) was used to determine the presence of lymphedema. Structured telephone interviews were conducted to assess postoperative sequelae and long-term complications.

**Results:**

Forty-three VFR patients (median age 63 years) were available for QoL assessment. Thirty-eight (88%) had received inguinal lymph-node dissection in addition to VFR. Mean global QoL (global health status) rating among all patients was 66.1 (± 25.5) on a scale from 0 to 100 with higher scores indicating better QoL. Higher GCLQ scores were significantly associated with lower global QoL scores (Spearman's rank correlation *ρ* =− 0.7, *p* < 0.0001). The presence of preoperative co-morbidities and postoperative wound-healing complications were also linked to reduced QoL (*p* < 0.01 for both). In a multivariable regression model, there was a significant interaction between preoperative co-morbidities and wound-healing complications with regard to global QoL (*p* < 0.05).

**Conclusion:**

Overall, VFR patients exhibit good quality of life postoperatively. The presence of lymphedema, wound-healing complications, and preoperative morbidities were associated with reduced QoL. Prospective longitudinal studies have to confirm our findings in the future.

**Electronic supplementary material:**

The online version of this article (10.1007/s00404-020-05584-5) contains supplementary material, which is available to authorized users.

## Introduction

Vulvar cancer accounts for about 5% of all malignancies of the female genital tract [1, 2]. Even though it is an uncommon cancer, its incidence has been increasing in recent decades [3, 4]. Whereas vulvar cancer is still regarded as a tumor of the elderly, the rising incidence has been driven largely by new cases among younger women [4, 5]. According to the Surveillance, Epidemiology, and End Results (SEER) database, 59% of patients have localized and another 29% regional disease at the time of diagnosis with 86.3% and 52.6% surviving for 5 or more years in each group, respectively [6]. Therefore, as most patients with vulvar cancer survive the disease, the number of years that patients live with treatment-related long-term complications and sequelae is increasing and post-treatment quality of life (QoL) deserves special consideration when counseling affected women about their treatment options. We have proposed vulvar field resection (VFR) as a novel approach to the surgical treatment of vulvar cancer [7, 8]. VFR is based on the theory of ontogenetic cancer fields which holds that malignant tumors infiltrate specific ontogenetically determined tissue domains in a stepwise and predictable manner. VFR is characterized by the local resection of a tumor within its ontogenetically specified field of potential growth (cancer field). While in some anatomic regions, this approach necessitates resection margins wider than 8 mm, other tissues directly abutting the tumor but belonging to a different ontogenetic domain can be preserved as they are not at risk for tumor involvement even when they are in close spatial proximity (< 8 mm). This facilitates optimal anatomic reconstruction by sparing important flap-donation tissues such as the labia majora in most cases and minimizes morbidity. Regional assessment for lymphatic tumor spread and therapeutic lymph-node dissection is another integral part of VFR. Adjuvant radiation is not administered either to the tumor field or the lymphatic drainage regions after VFR. We have already demonstrated excellent surgical and oncological outcomes achieved in the monocentric prospective Leipzig VFR trial [8]. In this trial, 97 consecutive patients were included of which 40% had lymph-node metastases and 36% had stage II disease or higher. Progression-free survival after 36 months was 85.1% and disease-specific survival was 86.0%. Here, we now describe patient-reported outcome measures evaluating QoL. Especially in light of the good survival outcomes, QoL is of great clinical importance and possibly more relevant than objective functional results after surgery. Because lymphedema of the lower extremities is one of the most prevalent and disturbing sequelae in vulvar cancer survivors, we here report its presence in VFR patients and investigate its correlation with QoL.

## Methods

### Study design

We performed a retrospective post hoc QoL analysis in patients who participated in the Leipzig School VFR study. The study was a prospective monocentric observational trial designed to evaluate the feasibility and surgical safety of VFR at the University of Leipzig’s cancer center [8]. Patients were eligible for participation if they were 18 years or older, had ontogenetic stage 1–3b vulvar cancer with or without lymph-node involvement, comprising FIGO stages I–III (more information regarding ontogenetic staging is available in the supplementary online resource p. 1 and Table S1 on p. 3) and had not undergone previous surgical or radiation therapy of the vulva. In addition, all patients were seen preoperatively by an anesthesiologist and had to be deemed fit for the operation. All patients provided written informed consent. Ethical approval was granted by the Leipzig University Institutional Review Board (156-2009-06072009 and 120-12-16042012). The trial (which was transformed into a multicentric study in February 2019) is registered at the German clinical trials registry (DRKS00013358). According to the above criteria, 97 consecutive vulvar cancer patients were included in the study between March 1, 2009, and June 8, 2017, the detailed surgical and oncological characteristics of this cohort have been published elsewhere [8]. A subset of this group consisting of all patients who were still alive in July 2017 and who were willing and capable to participate in QoL assessment as outlined below was included in the present investigation (Figure S1 in the supplementary online resource p. 4).

### Clinical and pathological data acquisition

All clinical and pathological data referred to in this investigation were collected prospectively as part of the Leipzig School VFR study. Medical co-morbidities were also assessed preoperatively by a gynecologic oncologist and an anesthesiologist as part of the study. All relevant information for the present investigation was later retrieved from the study records.

### Structured telephone interviews

All of the 94 patients who were still alive in July 2017 were contacted via telephone and submitted to a structured interview concerning long-term sequelae (including vulvar and perineal dysesthesia, problems with micturition and defecation, impaired pliability of the introitus, and subjective perception of vulvar symmetry). The complete structured interview questions are available in the supplementary online resource on p. 5.

### Quality-of-life assessment

Once a patient had completed the telephone interview and was physically and mentally capable of participating in QoL assessment, she was asked to complete the 30-item European Organization for Research and Treatment of Cancer Quality of Life Questionnaire (EORTC QLQ-C30) [9] which is currently one of the most commonly used and best validated instruments for patient-reported QoL assessment [10]. This questionnaire comprises five multi-item function scales (physical, role, emotional, cognitive, and social); three multi-item symptom scales (fatigue, nausea, and pain); six single-item symptom scales (dyspnea, insomnia, appetite loss, constipation, diarrhea, and financial difficulties); and one multi-item global QoL scale [9]. All raw scores are linearly transformed into a scale ranging from 0 to 100 with high values representing a higher functional level (EORTC QLQ-C30 functional scales) and high levels on symptom scales indicating the presence of more severe symptoms. Good-to-high reliability (Cronbach‘s alpha > 0.70) and good construct validity have been demonstrated for all scales of the German version [11].

### Patient-reported lymphedema assessment

We assessed the patient-reported presence of lymphedema using a German translation of the gynecologic cancer lymphedema questionnaire (GCLQ) [12]. The GCLQ is a 20-item questionnaire which evaluates the presence of patient-reported lower leg symptoms frequently associated with lymphedema (heaviness, swelling, presence of infections, aching, numbness, and physical functioning) during the past 4 weeks. Each of the 20 items is scored 1 or 0 and the total score is calculated by summation of all 20 items. Higher scores, therefore, indicate the presence of more severe lymphedema associated symptoms. Excellent internal consistency reliability has been reported for the English version of the questionnaire (Cronbach’s alpha = 0.95) [12].

### Assessment of sexual activity

In addition to the EORTC QLQ-C30 and the GCLQ, we asked patients whether they were sexually active using self-developed questionnaire items. If they were not engaging in sexual activity, we asked them to further specify the reason for their sexual abstinence using the following categories: lack of sexual partner, relationship problems, consequence of VFR treatment, or unspecified reasons which the patients did not want to disclose.

### Statistical analysis

All data were gathered and processed using Microsoft Excel (2016). Scoring of EORTC QLQ-C30 questionnaires was done in accordance with the current edition of the scoring manual [13]. Missing values were handled as outlined in the questionnaire guidelines. Scales were only analyzed when at least half of all relevant questions had been answered. In these cases, the mean of the answer of all items of the relevant scale was used as a substitute for missing values. For further statistical analysis, R [14] was used. We used non-parametric tests (Wilcoxon rank-sum test, Kruskal–Wallis test, and Chi-square test) to determine intergroup differences. Spearman’s rank correlation was used to calculate associations between GCLQ and EORTC QLQ-C30 scores. Categorical characteristics are reported as percentages, while medians and IQR are given for quantitative data. An exception to this is the reporting of EORTC QLQ-C30 scores when they are not subjected to further statistical testing. This is to facilitate the comparison with QoL data published elsewhere which is generally given as mean and standard deviation. We used linear regression analysis as implemented in the *glm* function of R [14] to investigate the interaction of preoperative co-morbidities and postoperative wound-healing complications in their association with global QoL.

## Results

The patient selection process is outlined in figure S1 of the supplementary online resource (p. 4). Of the 64 patients who could be contacted, 45 completed the questionnaire package as outlined in the methods section, yielding a response rate of 70.4%. Two patients had to be excluded from the study because they had received postoperative radiotherapy thus violating the study protocol (both patients had followed the recommendations of their general gynecologists who were providing follow-up care). Therefore, 43 patients were included in the final analysis. Forty-one EORTC QLQ-C30 forms were fully completed, while missing values had to be imputed in two cases as outlined in the methods section (one patient did not report on appetite and another omitted a question concerning role function). The median time elapsed between operation and patient assessment (follow-up) was 44 (IQR 25.7–69.7) months. Basic sociographic and clinical information are compiled in Table 1. The median age of our cohort was 63 years (IQR 56–75). Co-morbidities were present in 29 patients (67%). There were many advanced cases represented by the presence of nodal involvement in ten patients (23%), a median tumor size of 15 mm, and FIGO disease stage II or higher in nine patients (22%).

**Table 1:** Sociographic, patient, tumor, and treatment characteristics

Patients (*n* = 43)		
Socio-demographic characteristics (at the time of QoL assessment)		
Age, years (median, IQR)	63	56–75
Marital status-n, %		
Single	2	5
Married	22	51
Widowed	12	28
Separated	7	16
Educational status-n, %		
None	3	7
Primary	20	47
Secondary	13	30
Tertiary	7	16
Patient characteristics (at the time of QoL assessment)		
Body mass index, kg/m^2^ (median, IQR)	26	20–38
American Society of Anesthesiologists (ASA) score-n, %		
1	5	12
2	26	60
3	12	28
Comorbidities or risk factors-n, %		
Hypertension	20	47
Diabetes mellitus	6	14
Heavy smoking	1	5
Dementia or depression	2	5
Previous malignancies	4	9
None	14	33
Tumor characteristics		
Histological tumor type-n, %		
Squamous cell	42	98
Basal cell	1	2
Tumor size in mm-median, IQR		
Maximum diameter	15	10–34
Infiltration depth	5	3–12
Pathological tumor stage-n, %		
1a	5	12
1b	33	77
2	5	12
Ontogenetic tumor stage (oT)- n, %		
1	28	65
2	9	21
3a/3b	6	14
Pathological node stage-n, %		
0	33	77
1a	2	5
1b	3	7
2b	1	2
2c	4	9
International Federation of Gynecology and Obstetrics (FIGO) stage-n, %		
IA	5	12
IB	28	65
II	1	2
IIIA	4	9
IIIB	1	2
IIIC	4	9
Treatment characteristics		
Type of lymph-node dissection-n, %		
None	5	12
Sentinel	7	16
First-line inguinal	9	21
Total inguinal^a^	22	51
Type of anatomic reconstruction-n, %		
None (direct closure)	4	9
Random flaps	24	56
Axial pattern flaps	15	35
Selected complications/sequelae		
Postoperative inguinal wound infection leading to wound breakdown, stratified for nodal status-n, %		
pN0, n = 33	4	12.2
pN1, n = 10	4	40
Occurrence of postoperative lymphedema according to LNE type^b^-n, %		
No LNE	1	20
Sentinel LNE	2	28.6
First-line inguinal LNE	4	44.4
Total inguinal LNE	10	45.5

*IQR:* interquartile range

^a^including two patients with distal pelvic LND and one patient with inguinopelvic (lacunar) LND

^b^the number of patients receiving each LNE type is given above (treatment characteristics section of Table 1). See also Fig. S3A

### Quality of life

**Fig. 1:**
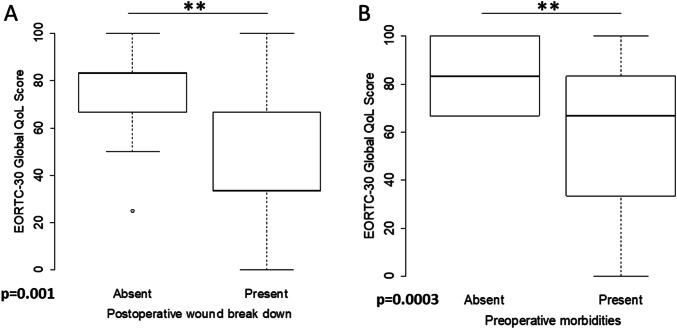
Factors associated with QoL. Boxplots indicating the distribution of global QoL scores stratified for the occurrence of postoperative wound breakdown (**a**) and the presence of preoperative morbidities as outlined in Table 1 (**b**)

The mean global QoL score was 66.1 (± 26.8). Of all EORTC QLQ-C30 function scales assessed, mean role functioning was lowest with 68.3 (± 33.9) points, while mean cognitive functioning was highest with 80.6 (± 26.7) points. Out of the three multi-item symptom scales, fatigue was on average rated highest with 34.8 (± 31.1) points, and out of the six single-item symptom scales evaluated with the EORTC QLQ-C30, sleep disturbance was highest with 28.7 (± 36.8) points (Table 2). There was a significant association between decreased global QoL with increasing patient age at the time of operation (*ρ* = − 0.3, *p* = 0.048). As determined by a Wilcoxon rank-sum test, the presence of preoperative co-morbidities and the occurrence of postoperative wound-healing complications were associated with significantly reduced global QoL (*p* = 0.0003 and *p* = 0.001, respectively, Fig. 1a, b). Multivariable linear regression analysis investigating the effect of preoperative co-morbidities and wound-healing complications on global QoL revealed a significant interaction between the two factors (*p* < 0.05, Table S2 in the supplementary online resource p. 4). The presence of overweight or obesity [body mass index (BMI) ≥ 25 kg/m^2^] was not linked to global QoL differences (*p* = 0.36,). The amount of time elapsed between operation and EORTC QLQ-C30 completion (< 48 months vs. ≥ 48 months) was also not associated with changes in QoL (*p* = 0.74). As determined by Kruskal–Wallis tests, none of the sequelae assessed in the structured telephone interview were linked to significantly reduced QoL (Figure S2 in the supplementary online resource p. 6).

**Table 2:** Results from QoL assessment with EORTC QLQ-C30

	Mean	Standard deviation
Multi-item function scales		
Physical functioning	73.5	25.6
Role functioning	68.3	33.9
Emotional functioning	75.9	27.8
Cognitive functioning	80.6	26.7
Social functioning	71.7	32.0
Multi-item symptom scales		
Fatigue	34.8	31.1
Nausea and vomiting	4.3	13.2
Pain	33.7	35.5
Single-item symptom scales		
Dyspnea	18.6	29.4
Sleep disturbance	28.7	36.8
Appetite loss	8.7	19.6
Constipation	11.6	25.1
Diarrhea	9.3	23.4
Financial impact	14.7	27.5
Multi-item QoL		
Global quality of life (global health status)	66.1	25.5

### Lymphedema

**Fig. 2:**
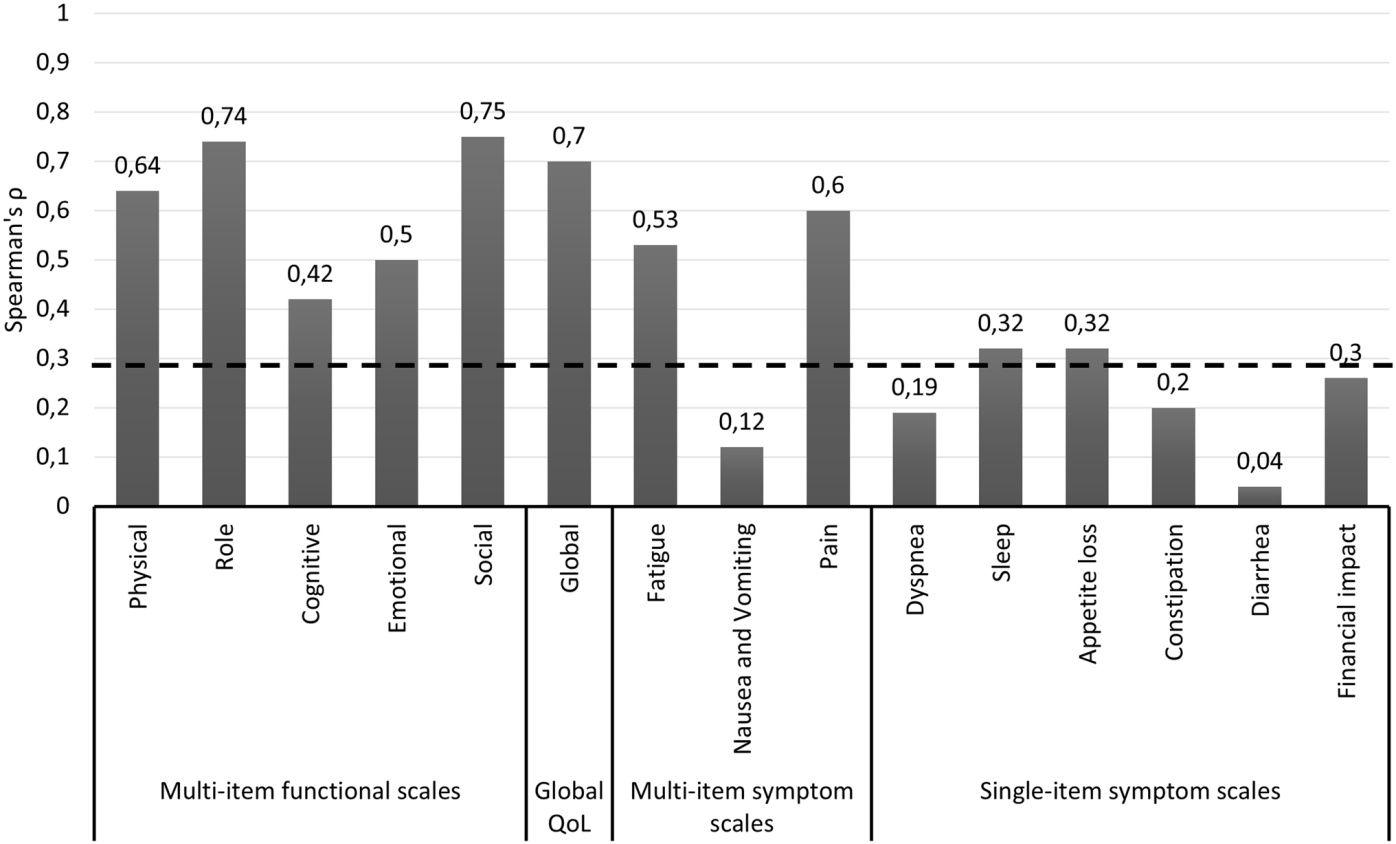
Correlation between GCLQ score and QoL. Results from Spearman’s correlation test evaluating the relationship between the scores of the GCLQ and the EORTC-QLQ-C30 scales. The dashed line indicates the significance threshold of *p* = 0.05. Higher *ρ* values indicate a stronger association

**Fig. 3:**
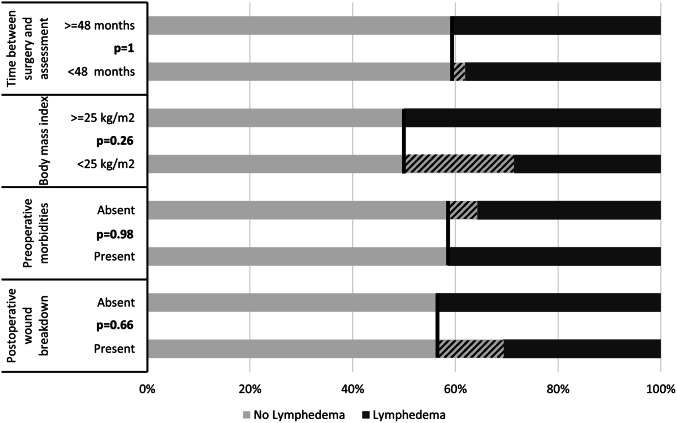
Factors associated with lymphedema. Barplots indicating the percentage of patients with lymphedema as determined by a GCLQ score of ≥ 5, stratified for different risk factors. The shaded plot segments highlight the size of the intergroup differences

The mean GCLQ score among all 43 patients in our study was 4.7 (± 4.7). Using a GCLQ score of ≥ 5 as cut-off value (yielding a positive and negative predictive value for the presence of lymphedema of 88.9% and 87%, respectively [12]), lymphedema was present in a total of 17 women (39.5%). Higher GCLQ scores were significantly associated with reduced QoL represented by worse outcomes on all five functional subscales (Fig. 2). Neither the presence of preoperative morbidities, a BMI of ≥ 25 mg/kg^2^, postoperative wound breakdown, nor a longer time since the operation (≥ 48 months) were significantly associated with changes in lymphedema symptoms as determined by Chi-square tests (Fig. 3).

### Sexual activity

**Fig. 4:**
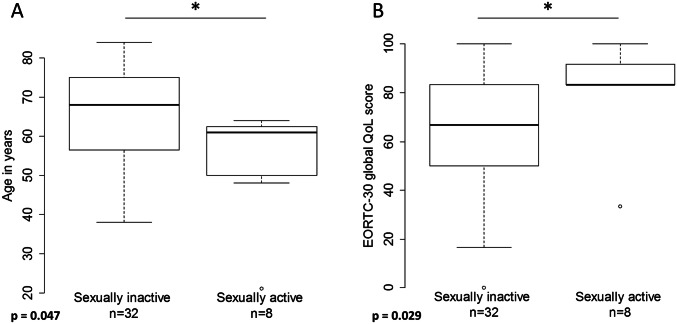
Correlation of global QoL with age and sexual activity. Boxplots indicating the age distribution of sexually active and inactive patients (**a**) and the distribution of global QoL scores among the same cohorts (**b**). Three patients did not disclose any information regarding sexual activity

Only 8 (18.6%) out of 43 patients reported to be sexually active and three patients did not disclose any information regarding sex life. Among the remaining 32 patients, the most common reason for sexual inactivity was the absence of a sexual partner (43.8%). Nine women (28.1%) reported that sexual activity was prohibited by sequelae of VFR. Six patients attributed their sexual inactivity to relationship problems unrelated to VFR (including medical problems of their partner) and three patients did not further specify the reason for their sexual abstinence. Among the 40 patients with available information, sexual activity correlated inversely with patient age and positively with global QoL (Wilcoxon rank-sum test, *p* = 0.047 and *p* = 0.029, respectively, Fig. 4).

### Anatomical reconstruction

All but four patients (9%) received anatomical reconstruction using local flaps (Table 1). The type of reconstruction used (direct closure, axial-pattern, or random-pattern flaps) was not related to postoperative QoL (Kruskal–Wallis test, *p* = 0.4345, Figure S4 in the supplementary online resource p. 7).

## Discussion

In this study, we present for the first time results from QoL assessment in patients who underwent VFR with therapeutic lymph-node dissection and anatomical reconstruction. As VFR exhibits excellent cure rates [8], postoperative QoL is of great relevance. In several aspects, QoL in our patients was not different from that in a representative German reference population of women without cancer. For example, most of our study patients were 60 years of age or older (*n* = 28, 65%). Even though we observed a statistically significant decline in global QoL status with increasing patient age, mean global QoL in the subgroup of patients aged ≥ 60 years was still 63.5 (± 24.2). In the German reference population of healthy women [15], the mean global QoL score was 69.0 for subjects aged 60–69 years and 60.2 for women aged more than 70 years.

Comparison of our QoL data with that from other studies involving vulvar cancer patients is limited because of different patient selection criteria, and varying study protocols (e.g., time of QoL assessment), and small-sample sizes impeding subgroup analyses. Keeping these limitations in mind, nevertheless, a cautious comparison with some studies can be made. For example, Hellinga et al. [16] report a mean global QoL score of 75.76 in a group of 22 patients who were treated for (pre-)malignant lesions of the vulvoperineal area surgically and who underwent reconstruction using a lotus petal flap. In their cohort, however, the patients appeared to be somewhat healthier at the outset of treatment as suggested by the absence of any patient with an ASA score higher than 2. In contrast, 28% of the patients in our group were scored ASA 3, and our data demonstrate that preoperative morbidities are associated with reduced postoperative QoL. Moreover, 54% of the patients in their study were not treated for vulvar cancer but for other disease entities. In another study, Novackova et al. [17] compared QoL in vulvar cancer patients treated surgically with SLNB (CON group) or with inguinofemoral lymphadenectomy (RAD group). Global QoL was 70.5 and 72.5 after 6 and 12 months, respectively, in the SLNB group, while it was 64 and 65 after 6 and 12 months in the inguinofemoral lymphadenectomy group. Additionally, 50% of the RAD group received radiotherapy due to groin node metastasis. The QoL of the RAD group was only slightly worse in comparison to our findings. This might be attributable to the median number of lymph nodes removed per groin. The median number in the RAD group was 4.7 (range 3–6), whereas it was 8 (range 0–17) in our study. Also, there was no information regarding whether the RAD group was treated with local excision or modified radical vulvectomy. These differences in treatment and extent of (inguinal) and vulvar surgery clearly impede comparison.

An important result was the clear association between the preoperative presence of medical co-morbidities and reduced postoperative QoL (*p* = 0.0003, Fig. 1b). Even though this is not unexpected, it is of special relevance in our study as it can serve as a proxy for preoperative QoL. For example, arterial hypertension [18] and diabetes mellitus [19] have both been shown to negatively impact QoL in large studies. Therefore, it is not unlikely that a significant number of patients with low EORTC QLQ-C30 scores postoperatively had already been suffering from lower QoL before surgery.

Importantly, we found that wound-healing complications were also associated with reduced global QoL, with a significant interaction observed with the presence of preoperative co-morbidities in a multivariable linear regression model. The decreased postoperative global QoL in patients who had wound-healing complications is, therefore, probably best explained by their preoperative constitution rather than by immediate effects of secondary wound healing. This is in accord with our previous findings, indicating that long-term functional and cosmetic outcomes are not inferior in patients who experience wound-healing complications as compared to those who do not have such problems [8]. We did not observe an effect of the time interval elapsed since surgery and quality of life. Interestingly, a recent longitudinal study observed a significant decrease in several QoL scales [20]. In that study, almost 30% of patients received adjuvant radiation and the authors attributed the worsening QoL at least partially to side effects of radiotherapy. VFR does not necessitate adjuvant radiation even in the presence of risk factors and this clear advantage over the conventional therapy might contribute to stable QoL postoperatively. There was also no association between various postoperative sequelae assessed in the structured interviews and QoL (Figure S2 in the supplementary online resource p. 6). This finding is probably best explained by the so-called “response shift” observed in QoL investigations involving cancer patients. This concept describes how QoL in cancer survivors is better than expected in light of treatment sequelae and long-term symptoms, because these patients experience a change in their frame of reference thereby reconceptualizing, reprioritizing, and recalibrating different psychological and physical aspects contributing to overall QoL [21, 22].

The presence and severity of symptoms related to lymphedema as assessed by the GCLQ were significantly associated with reduced QoL (Fig. 2), a finding which has been reported by others [17, 23–27]. Interestingly, the type of lymph-node dissection (sentinel LND, first line LND, or total inguinal LND) was not related to the degree of either lymphedema symptoms or QoL (Figure S2 in the supplementary online resource p.6). The latter aspect of this finding is in accord with data from a larger study by Oonk et al., reporting that there was no difference in terms of QoL between patients undergoing sentinel LND and total inguinal LND [28]. Even though there was a trend to less lymphedema in patients who received sentinel LND as compared to those who received first line or total inguinal LND, this difference was not significant, probably because of our small-sample size (Figure S3 in the online resource p. 7). It should be noted that comparison of our lymphedema findings with results from other trials is limited, because generally different assessment methods are used. Lymphedema is commonly evaluated by clinical observation, assessment of pitting, measurements (e.g., leg circumference or water displacement measurements), and bioimpedance spectroscopy. The GCLQ, on the other hand, assesses patient-reported symptoms. While patient-reported symptoms seem more relevant than clinical signs of lymphedema, GCLQ scores might also overestimate the incidence of true lymphedema. This is supported by a recent study involving lymphedema assessment in 30 vulvar cancer patients in which the investigators demonstrated that self-reported lymphedema was present in 12/18 patients (67%), while it could be detected by bioimpedance measurements in 1/12 (8.3%) only [29]. Other studies, however, have shown that clinical findings and GCLQ scores correlate reasonably well [12, 30, 31].

Only 18.6% of our patients reported to be sexually active. This number corresponds well with the results from other studies reporting sexual activity in 17.7% [28] and 21.4% [23]. In the latter study, the investigators demonstrated that there was no difference in sexual activity in a comparable cohort of healthy patients. In a comprehensive review [32], Aerts et al. concluded that besides a history of depression and excision size of the vulvar lesion, poor overall QoL and patient age were the most important determinants of sexual activity, a finding which our study confirms (Fig. 4). Importantly, both QoL and sexual activity are significantly influenced by age. In a study by Grimm et al. reporting QoL and sexual functioning of 21 vulvar cancer patients undergoing conventional surgical treatment, 38.1% of the women were sexually active [33]. However, the median patient age in that study was 52 years as compared to 63 years in our study. Interestingly, out of 12 patients who specified the reason for their sexual inactivity, eight (66.7%) stated physical problems related to the surgery interfering with sexual intercourse [33]. In our study, only 28.1% of patients attributed their sexual inactivity to VFR sequelae.

To our knowledge, this is the largest cohort of anatomically reconstructed patients of whom QoL outcomes are reported. As has been shown before, anatomic reconstruction is an important determinant of good functional status and cosmetic outcome [34–36]. However, up to date, there have been no prospective studies evaluating the impact of different reconstructive techniques or anatomical reconstruction in general on QoL. One recent retrospective analysis including 12 vulvar cancer cases who received reconstruction using pedicle flaps reported a mean global QoL score of 75.7 [16]. This number cannot be compared to our collective, however, because no information regarding regional treatment was available and the sample size is small. In our patients, we did not observe a difference in QoL between the different types of flaps used (Figure S4 in the supplementary online resource p. 7).

Our study has several important weaknesses. First, it is a single-group observational study and the results can only be compared to historic controls from similar cohorts preventing any inferences on causality and all analyses have to be considered exploratory. Second, the generalization of our findings is restricted by the small-sample size. This, however, is a common limitation in vulvar cancer studies as it is a rare—although increasing—entity. Third, our study design did not include preoperative QoL assessment which prohibits any definitive statement regarding the effect of VFR on QoL over time. This will be determined by the results from our ongoing prospective multicentric VFR study. Yet, our data demonstrate that VFR in addition to leading to excellent oncological outcomes is also associated with good postoperative QoL. For the first time, we demonstrate that postoperative wound-healing complications and the presence of preoperative morbidities are associated with significantly impaired QoL. As there is an interaction between these two factors, the presence of preoperative medical comorbidities seems to identify a patient collective both at risk for wound-healing disturbances and reduced quality of life. Further studies including our own ongoing multicentric VFR trial need to corroborate these findings.

## Electronic supplementary material

Below is the link to the electronic supplementary material.

Supplementary file1 (DOCX 1160 kb)
