# Supplementary material for: Quality of life and associated factors after surgical treatment of vulvar cancer by vulvar field resection (VFR)
Source: Arch Gynecol Obstet. 2020 May 19;302(1):191–201. doi: 10.1007/s00404-020-05584-5 (PMC7266857; doi:10.1007/s00404-020-05584-5)
Supplement: Supplementary file 1 — Supplementary file1 (DOCX 1160 kb) [file 404_2020_5584_MOESM1_ESM.docx]

**Electronic Supplementary Material**

## **Quality of life and associated factors after surgical treatment of vulvar cancer by vulvar field resection (VFR)**

Sophia Trott^1^, Michael Höckel MD PhD^1^, Nadja Dornhöfer MD^1^, Kristina Geue PhD^2^, Bahriye Aktas MD^1^, Benjamin Wolf MD^1^*

## **Ontogenetic tumor staging of the vulva**

Details on the ontogenetic cancer field resection theory applied to vulvar cancer in the form of ontogenetic staging and vulvar field resection have been published and can be found elsewhere [1]. Here, only a very brief overview over ontogenetic staging will be given. Cancer is thought to propagate in ontogenetically defined tissue domains (cancer fields) and progresses from one domain into the next in a stepwise and predictable fashion. This stepwise progression is reflected in ontogenetic tumor staging. A cancer growing within one vulvar sub-compartment which constitutes the mature derivative of a common embryonal immediate precursor tissue is staged oT1. Once progression into one or more neighboring vulvar sub-compartment occurs, the tumor is staged oT2. If tissues are involved which are not part of the vulvar proper and are therefore derived from more distant precursor-tissues they are staged oT3a, oT3b and oT4. The specific mature tissues constituting one ontogenetic domain are summarized in **table S1** on page 3.

## **Lymph node dissection**

The techniques of lymph node dissection have already been published [1]. Here, only a brief definition of the LND techniques mentioned in the main text will be given. For more information please refer to the indicated publication.

Sentinel LND

For this procedure, which is only performed in patients with oT1 disease, women are injected with radio-active Technetium 99^m^ around the tumor on the evening before the operation. Intraoperatively the sentinel-node is detected using a radioactivity detection probe. 1-2 sentinel nodes are removed per groin.

First line inguinal LND and total inguinal LND

After a curved incision over the presumed entry point of the great saphenous vein into the femoral vein, the femoral triangle is delineated by identifying the inguinal ligament cranially, the Sartorius muscle laterally, and the adductor longus muscle medially. It is the divided into four quadrants by a line following the leg axis and a second line intersecting at a 90° angle at the entry of the great saphenous vein into the femoral vein. This way, the superolateral, superomedial, inferomedial, and inferolateral superficial inguinal subregions are defined. The deep inguinal lymph nodes are located below the fascia lata medially to the proximal femoral vein within a small pit formed by the adductor longus and pectineus muscles. First line LND dissects the lymph nodes in the superomedial, superolateral and inferomedial quadrants. Total inguinal LND comprises removal of the lymph fatty tissue of the inferolateral quadrant and the deep inguinal lymph nodes in addition to the three first line quadrants.

Inguinopelvic (lacunar) and distal pelvic LND

The inguinopelvic (lacunar) nodes are located proximal (cranial) of the inguinal ligament in the lacuna vasorum and are removed when second line lymph nodes contain metastasis (two patients in our cohort). The distal pelvic lymph nodes are even more cranial surrounding the external iliac and obturator vessels in the pelvis. These are removed when metastasis is present in the lacunar nodes (one patient in our cohort).

| **Ontogenetic tissue domain** | **Anatomical structures** | | **oT stage** |
| --- | --- | --- | --- |
| Peripheral subcompartment | Praeputium clitoridis | | oT1 |
|  | Sulcus interlabialis | |  |
|  | Lateral perineal skin | |  |
| Intermediate subcompartment | Glans clitoridis | | oT1 |
|  | Frenula clitoridis | |  |
|  | Labia minora | |  |
|  | Medial perineal skin | |  |
|  | Ventral segment of the anal skin (between 11 and 1 o'clock) | |  |
| Central subcompartment | Vestibulum vaginae | | oT1 |
|  | Meatus urethrae | |  |
|  | Outer hymenal rim | |  |
|  | Vestibular glands | |  |
| Vulvar compartment | Structures of the peripheral, intermediate, and central subcompartments | | oT2* |
| Vulvar metacompartment | Structurs belonging to the vulvar subcompartments **AND** | | oT3a |
|  | Erectile tissues (Corpus et crura clitoridis, Bulbus vestibularis) | |  |
|  | Distal urethra | |  |
|  | Distal vagina | |  |
| Urogenital plate metacompartment | Structures belonging to the vulvar compartment **AND** | | oT3b |
|  | Fascia and fatty tissue belonging to the labia maiora (Dartos) | |  |
|  | Perianal skin | |  |
|  | Urinary bladder mucosa | |  |
|  | Fascia inguinalis | |  |
|  | Fascia pubica | |  |
|  | Septum anovestibulare (central perineal body) | |  |
| Clocal membrane metacompartment | Structures belonging to the late vulvar metacompartment **AND** | | oT4 |
|  | Ligamentum pubourethrale |  | |
|  | Abdominal and pelvic wall |  | |
|  | Lower extremities |  | |
|  | Genital tract |  | |
|  | Unrinary tract |  | |
|  | Muscles and fascia of the perineum and pelvic floor |  | |
| *Tumor involves more than 2 subcompartments | Anus and rectum |  | |

**Table S1: Ontogenetic anatomy of the vulva as the basis for oT staging**

**Figure S1: Patient selection process**


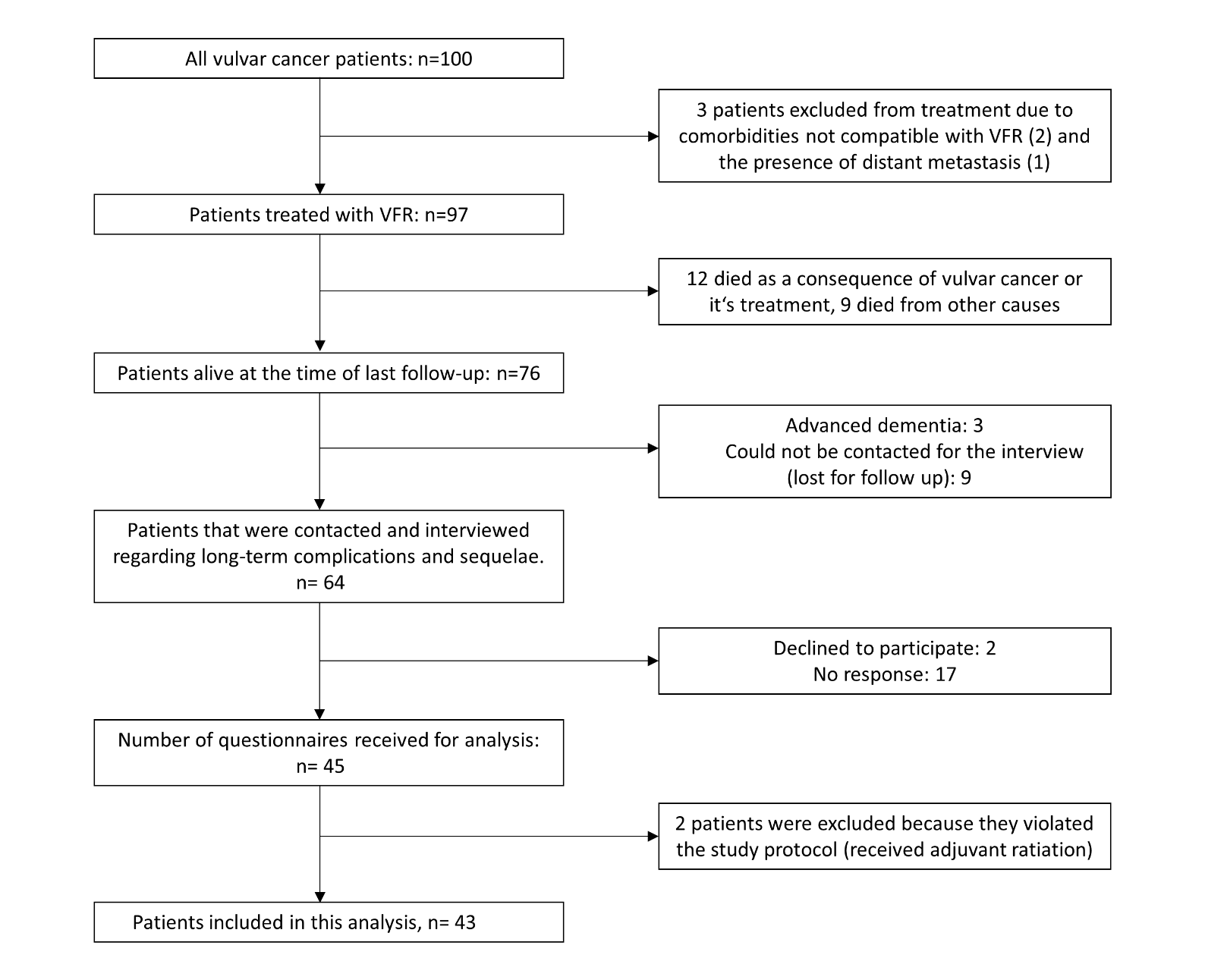


**Table S2: Multivariable linear regression modelling (effect on global QoL)**

|  | Regression coefficient | Standard error | T-value | p-value |
| --- | --- | --- | --- | --- |
| Intercept | 84.556 | 6.125 | 13.805 | <0.0001 |
| Age (years) | -0.1765 | 0.2259 | -0.781 | 0.4395 |
| Woundhealing complications present | 8.5802 | 13.9183 | 0.616 | 0.5413 |
| Preoperative comorbidities present | -14.3893 | 6.818 | -2.110 | **0.0415** |
| Interaction between woundhealing complications and preoperative comorbidities | -38.7201 | 15.5414 | -2.491 | **0.0172** |

# **Structured questionnaire for the assessment of postoperative morbidity and sequelae**

When not otherwise specified, the following questions pertain to the last four weeks.

All items are rated on the following scale: “not at all” – “a little bit” – “somewhat” – “very much”

1. Do you feel a lack of your bodily femininity resulting from the operation?
2. Do you feel that your vulva/pubic area has been mutilated by the operation?
3. Does your new vulva/pubic region deviate from a symmetrical appearance?
4. Do you realize impairment of sensibility around the reconstructed vulva?
   1. Numbness
   2. Tingling
   3. Pain
   4. Itching
5. Do you realize impairment of sensibility around the groin scars? If yes: on which side?
   1. Numbness
   2. Tingling
   3. Pain
   4. Itching
6. Do you realize impairment of sensibility in the flap-donation area? If yes: on which side?
   1. Numbness
   2. Tingling
   3. Pain
   4. Itching
7. Do you have difficulties with voiding?
   1. Do you experience involuntary loss of urine?
   2. Do you have to empty your bladder using catheters?
   3. Do you have pain during voiding?
8. Do you have difficulties with bowel movement?
   1. Do you have constipation?
   2. Do you experience involuntary loss of stool?
   3. Do have pain with bowel movement?
9. Do you notice a swelling?
   1. In the right leg?
   2. In the left leg?
   3. In the right groin?
   4. In the left groin?
   5. In the mons pubis area?
10. Do you undergo lymphatic drainage/massage?

(To be answered on the scale “no” – “once per month or sometimes” – “once per week or regularly” – “more than once per week”)

1. Since the operation, have you observed reddening of the skin around the vulva, groin or legs which was treated with antibiotics?

(To be answered on the scale “never” – “once” – “twice” – “more than twice”)

Note: this questionnaire has been published before with the results from the VFR-trial [1].

**Figure S2:**


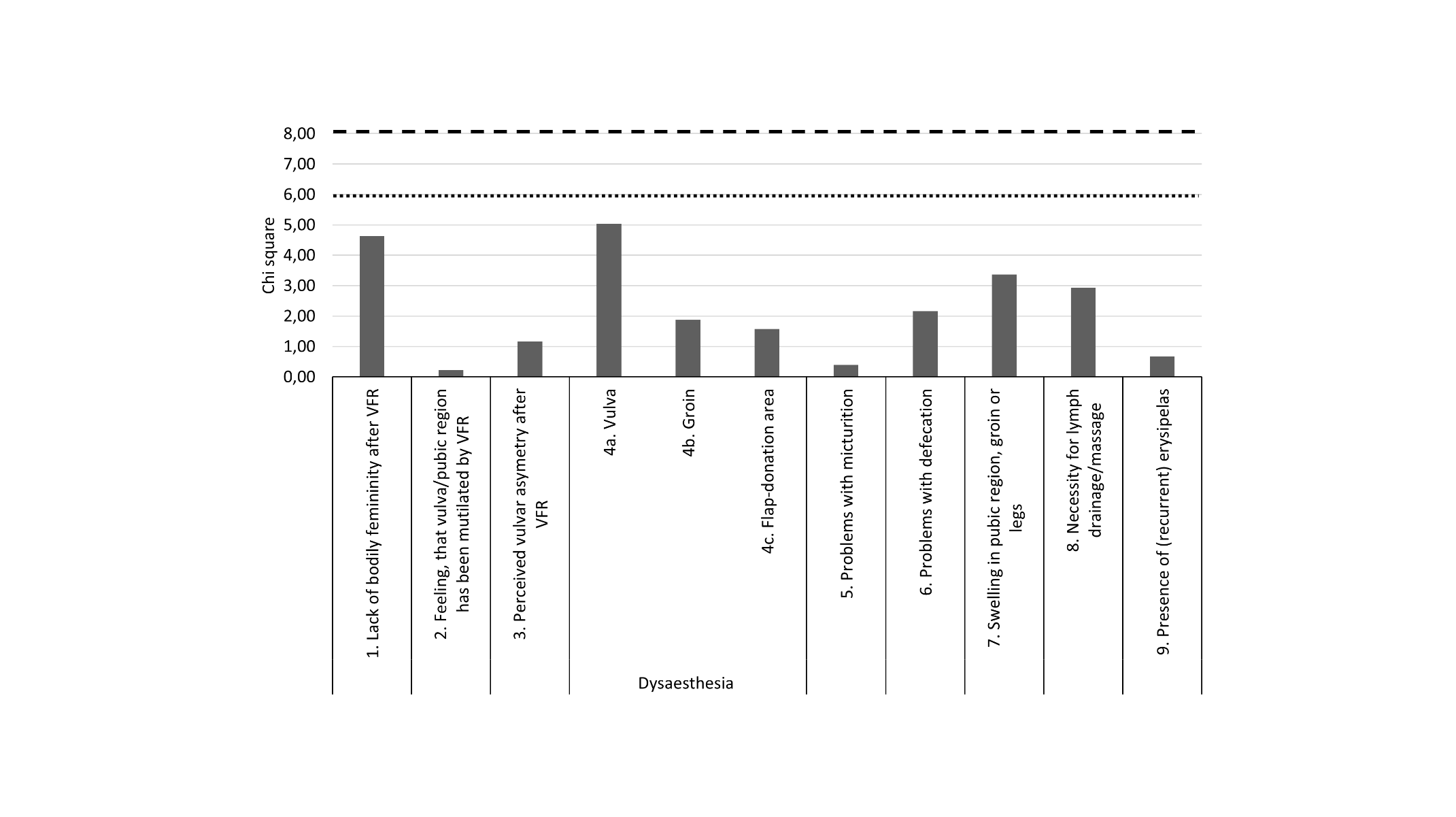


**Figure S2:** Association between global QoL and postoperative sequelae as assessed by the structured telephone interviews. Chi square values calculated with the Kruskal-Wallis test are shown. The dotted and dashed lines indicate the p=0.05 significance level for 2 and 3 degrees of freedom, respectively (3 degrees of freedom for questions whose responses were rated in 4 categories and 2 degrees of freedom for responses with 3 response categories,

# **Figure S3:**


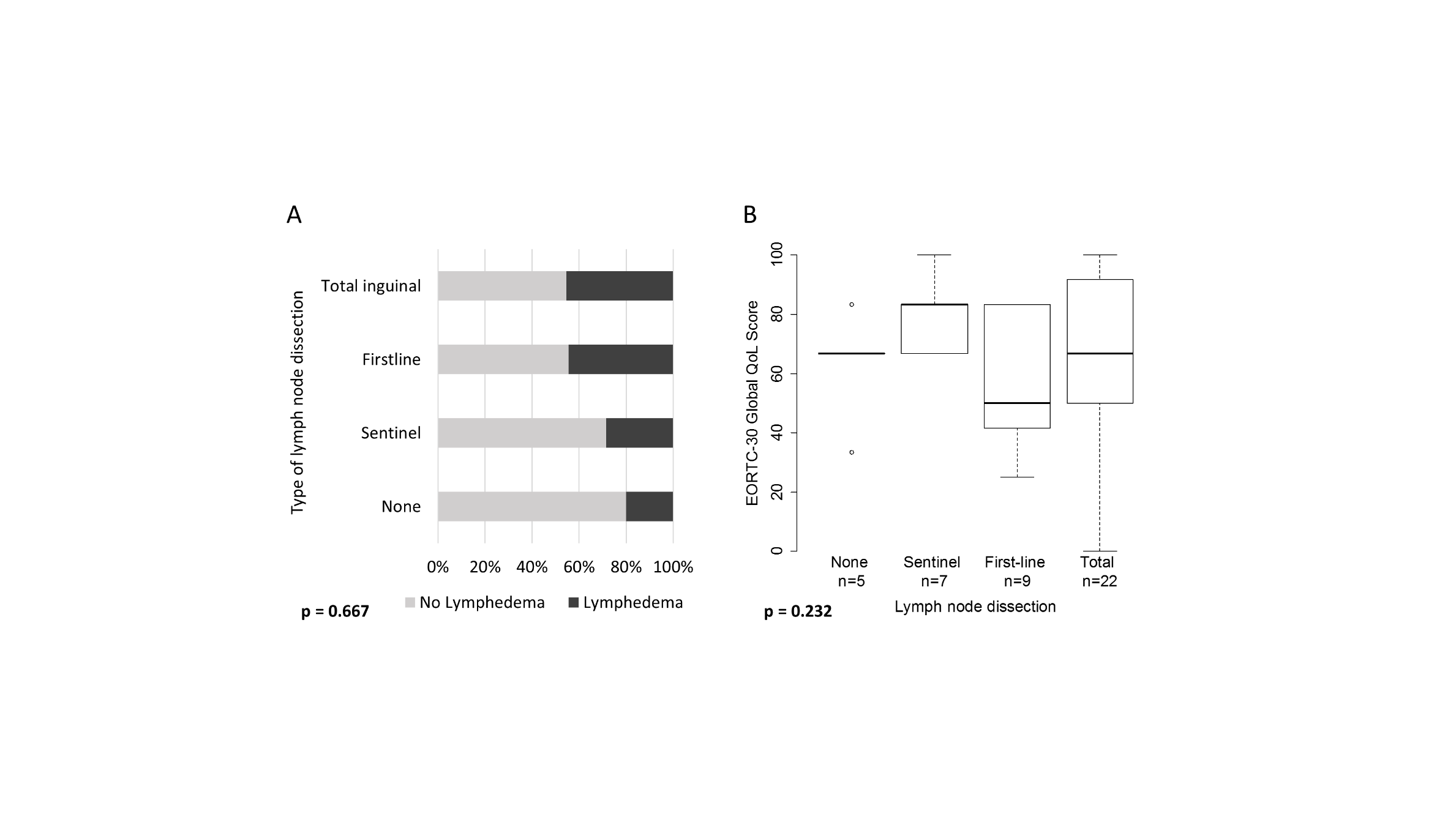


**Figure S3:** **A**: Association between the presence of lymphedema as determined by a GCLQ score of ≥ 5 and type of lymph node dissection are shown (not significant by Chi squared test with 3 degrees of freedom, p=0.667). **B:** Boxplots indicating the distribution of global QoL scores stratified for type of lymph node dissection. No statistically significant correlation between the type of lymph node dissection and global QoL could be found (p=0.232).

**Figure S4:**


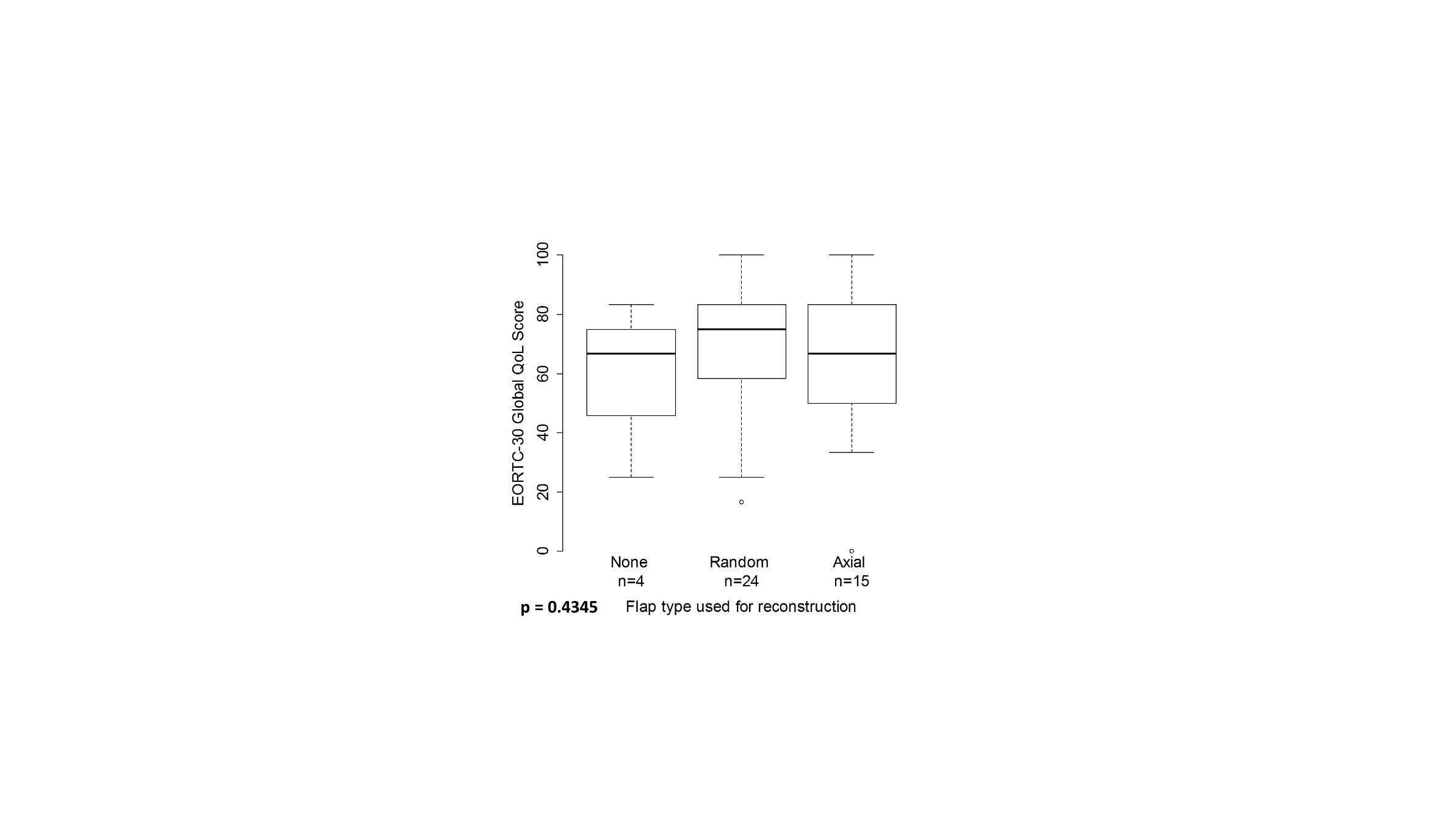


**Fig. S4:** Boxplots indicating the distribution of global QoL scores stratified for type of anatomical reconstruction used. There is no statistically significant association as determined by a Kruskal-Wallis test (p=0.4345).

**References**

1. Höckel M, Trott S, Dornhöfer N, et al. Vulvar field resection based on ontogenetic cancer field theory for surgical treatment of vulvar carcinoma: a single-centre, single-group, prospective trial. *The Lancet Oncology* 2018;4:537–548.
